# A Secondary Analysis of Caloric Restriction and Exercise Effects on Cognitive Function in Functionally Limited Postmenopausal Women with Overweight or Obesity

**DOI:** 10.3390/nu17132075

**Published:** 2025-06-22

**Authors:** Christian W. McLaren, Rebecca L. Pearl, Glenn E. Smith, Stephen D. Anton

**Affiliations:** 1 Department of Clinical and Health Psychology, College of Public Health and Health Professions, University of Florida, Gainesville, FL 32611, USA; mclaren@ufl.edu (C.W.M.);; 2 Department of Physiology and Aging, College of Medicine, University of Florida, Gainesville, FL 32611, USA

**Keywords:** cognition, lifestyle, obesity, postmenopausal, women, calorie restriction, exercise, randomized controlled trial

## Abstract

Background: Postmenopausal women face a higher risk of obesity and related chronic diseases. While lifestyle interventions improve cardiometabolic health and physical function, their effects on cognitive function remain understudied, especially in diverse populations. This study examined the impact of a lifestyle intervention combining caloric restriction and exercise on cognitive function in a diverse sample of postmenopausal women with overweight or obesity and functional limitations. Methods: This study represents a secondary analysis of a previously conducted pilot trial, in which 34 participants were randomly assigned to a 24-week intervention: (i) caloric restriction plus exercise (CR + E; n = 17) or (ii) educational control (EC; n = 17). In the CR + E group, participants engaged in group-based weight management focused on caloric restriction and three weekly exercise sessions, including walking and lower-body resistance training. The EC group attended monthly health education lectures. Changes in cognitive scores were assessed using the Digit Symbol Substitution Test (DSST) and the Controlled Oral Word Association (COWA) test. Additionally, we explored the correlation between changes in cognitive scores and physical function in the CR + E group. Results: In the CR + E group, DSST scores significantly improved compared to the EC group (*p* < 0.05). There were no significant changes in COWA scores for either group compared to their baseline value or between groups. Furthermore, changes in DSST or COWA were not significantly correlated with changes in walking speed or physical function. Conclusions: The preliminary results of this study suggest that CR + E may improve complex attention in functionally limited postmenopausal women with overweight or obesity but does not appear to significantly affect verbal fluency.

## 1. Introduction

Projections in the United States have indicated a significant demographic shift, with the population of adults aged 65 and older expected to double by 2050 [1]. This growth in older adults is anticipated to be accompanied by an increase in the prevalence of those fitting the criteria for overweight or obesity, given recent trends. Specifically, in 2007–2010, 35% of older adults met the criteria for obesity (BMI ≥ 30 kg/m^2^), increasing to 42.8% by 2017–2018 [2,3]. In addition to these trends, there are noted discrepancies. Specifically, women aged 65 years and older have a higher prevalence of obesity than men, and those who identify as non-Hispanic Black women face the largest disparity, having 1.75 times higher odds of class 2 (BMI 35–39.9 kg/m^2^) and class 3 (≥40 kg/m^2^) obesity compared to non-Hispanic White women [4].

The rising prevalence of obesity in high-risk populations is concerning due to the association of numerous chronic health conditions with obesity, including type II diabetes and cardiovascular disease [5]. Obesity also appears to increase the risk of dementia in later life [6,7]. A recent study found that individuals with overweight or obesity (BMI ≥ 25 kg/m^2^) in mid-and late life had a faster rate of cognitive decline and a higher Alzheimer’s risk than those with lower BMIs (18.5–24.9 kg/m^2^) [8]. In addition, obesity in older adulthood has been linked to an increased risk of functional decline, which can in turn lead to an increased risk of disability and a reduced capacity for independent living [9]. Recent research suggests a relationship between physical and cognitive function, with lower Short Physical Performance Battery (SPPB) scores being correlated with lower Mini-Mental Status Examination (MMSE) [10]. Notably, physical activity may help mitigate the potential negative effects of obesity on cognition, as higher physical activity levels are associated with improved cognitive outcomes, including global cognition, memory, and verbal fluency, even at modest activity levels [11,12].

In addition to biological and behavioral factors influencing cognitive decline, social and psychosocial factors play a critical role in cognitive aging, particularly for individuals with obesity, who face increased risks of stigma, social isolation, and mental health challenges. Weight-related stigma is linked to depression, anxiety, emotional eating, reduced physical activity, and poor sleep, all of which may contribute to cognitive decline [13,14]. Social isolation and loneliness further exacerbate the risk of dementia [15,16], with lower social support correlating with greater distress in this population [17]. Conversely, social engagement through structured group activities supports cognitive health by providing mental stimulation, reducing stress, and fostering emotional well-being [18,19]. Thus, group-based interventions that provide social opportunities may enhance cognitive function beyond the effects of weight loss or exercise alone.

Studies have demonstrated that group-based interventions that utilize caloric restriction or exercise independently yield beneficial effects on both physical and cognitive outcomes in middle-aged and older adults [20,21]. However, when caloric restriction and exercise are combined there are synergistic effects, leading to a significant reduction in weight and improvements in physical functioning in older adults with obesity [22,23]. Moreover, results from studies utilizing combined caloric restriction and exercise have shown promise in enhancing aspects of cognitive function, including executive functioning and processing speed in middle-aged and older adults [24,25,26]. While these findings are promising, a limitation of previous trials is their heterogeneity, with many studies primarily including those who identify as non-Hispanic White and female. As a result, there is limited information on the effects of these interventions on racially diverse samples, particularly Black women, who are at heightened risk for obesity-related complications.

For these reasons, the primary aim of this secondary analysis was to assess the effects of a group-based caloric restriction plus exercise (CR + E) intervention on measures of cognitive function in a sample of non-Hispanic Black and White postmenopausal women with overweight or obesity and functional limitation (SPPB score between 4 and 10), compared to an educational control (EC). Additionally, for exploratory analysis, we also aimed to determine potential associations between changes in cognitive function and changes in physical activity. We hypothesized that: 1) the CR + E intervention would enhance the cognitive outcomes studied, compared to an EC group; and 2) that participants randomized to the CR + E group would show a positive association between changes in physical function and changes in cognitive function.

## 2. Materials and Methods

This secondary analysis utilized data from the weight loss plus exercise pilot randomized controlled trial, which primarily aimed to assess changes in physical function and weight following a 6-month participation in a CR + E intervention in sedentary, postmenopausal women with overweight or obesity and functional limitations compared to an educational control group [22]. The parent study showed that participants randomized to the CR + E group experienced significantly greater weight loss, increased walking speed, and improved SPPB scores compared to the EC group.

While the parent study focused on physical function and weight loss, the purpose of this secondary analysis was to examine whether the CR + E intervention led to significant changes in cognitive function from pre- to post-intervention. Additionally, we explored the potential associations between changes in cognitive function and changes in physical function in the intervention group.

### 2.1. Study Design and Participants

For detailed methodology, please refer to the original publication by Anton et al. [22]. Briefly, in the parent study, participants were recruited via mailers and flyers. Those who expressed interest completed a telephone screening, and those who were still eligible for the study and were still interested were invited for an in-person screening visit to further assess eligibility criteria. Main study inclusion included those aged 55–79 years old, BMI > 28, non-Hispanic Black or White racial identity, and self-reported sedentary behavior. Those still eligible, following the in-person screening, were randomized to either the CR + E or EC group and were invited back for a baseline assessment visit, during which anthropometric, cognitive, and physical functioning measurements were obtained. Following the 24-week intervention, measurements were reobtained.

As previously described, the CR + E pilot trial included 34 sedentary, postmenopausal women with overweight or obesity and mild to moderate functional limitations, as measured by a total SPPB score ranging from 4 to 10 [22]. This study was approved by the University of Florida Institutional Review Board, and all participants completed informed consent procedures and agreed to take part in the study.

### 2.2. Experimental Groups

Participants were randomly assigned to one of two groups for the six-month study: The CR + E group followed a daily 750 kcal deficit, determined by baseline food log evaluations conducted by a study dietitian, and adhered to a diet consisting of 55% carbohydrates, 30% fat, and 15% protein based on American Heart Association recommendations [27]. They attended weekly dietary group sessions to review food logs and receive individualized weight-loss recommendations and participated in three weekly 60 min moderate-intensity, group-mediated exercise sessions, which included a 15-min aerobic warm-up, 15-min of lower-body resistance exercises, 15-min of brisk walking, and a 5 min flexibility cooldown. Group sessions were monitored by a trained study interventionist to provide standardized recommendations for exercise intensity and technique. Participant exercise intensity was monitored utilizing the Borg Rating of Perceived Exertion (RPE) Scale, utilizing a scale ranging from 6 to 20 [28]. After a three-week ramp-up period, participants were instructed to perform walking at a moderate intensity (13 RPE) and lower-body resistance exercises at a vigorous intensity (15–16 RPE). Beginning in the third week, participants in the CR + E group were encouraged to achieve a weekly goal of 150 min of walking. The EC group was instructed to maintain their current exercise and dietary habits and attended monthly educational lectures on topics unrelated to diet or exercise.

### 2.3. Outcomes

#### 2.3.1. Cognitive Measures

Digit Symbol Substitution Test (DSST): The DSST evaluates complex attention and serves as a marker for psychomotor speed, working memory, and visual-perceptual functioning [29]. Participants match symbols to numbers within a grid of 100 boxes, using a provided key. A sample trial precedes the assessment to ensure comprehension. Participants then complete as many boxes as possible within 90 s, aiming to match each number with its corresponding symbol without skipping any. Scoring involves tallying correctly paired symbols, yielding the total score.

Controlled Oral Word Association Test (COWA): The COWA test, also known as “FAS,” assesses language and verbal fluency [30]. Participants generate words starting with designated letters (F, A, S) within 60 s, avoiding proper nouns, numbers, slang, or repeated words with different suffixes. Scores are based on the total number of eligible words across three-letter trials.

#### 2.3.2. Physical Measures

Short Physical Performance Battery (SPPB): The SPPB evaluates physical functioning through three domains: balance, walking speed, and lower extremity strength. Balance is assessed using three different standing tests: side-by-side, semi-tandem, and tandem stance, with participants needing to hold each position for 10 s to achieve maximum points. Walking speed is measured by timing participants as they walk a 4 m distance at their normal pace. Lower extremity strength is evaluated by timing participants as they complete five sit-to-stand repetitions. Scores from each domain are calculated and summed to yield a total score.

The 400 Meter-Walking Test (400MWT): 400MWT is used to determine typical walking speed. The testing course is set up using two cones placed 20 m apart. Participants are instructed to walk at their normal pace to one cone, pivot, and walk back, repeating this process for a total of ten laps. Participants are advised not to overexert themselves and are allowed to stand in place to catch their breath if needed. The total distance walked, and the time taken to complete the test are recorded. Average walking speed is calculated by dividing the total number of meters walked by the time taken to complete the test.

### 2.4. Statistical Methods

This was a secondary analysis of a previously conducted pilot study. Consequently, the study was not powered for the analyses of cognitive outcomes. A post hoc power analysis using G*Power (version 3.1.9.7) assessed the statistical power for differences in change scores between the CR + E and EC groups on the DSST and COWA [31]. The computed power (1-β) was 0.72 for DSST (d = 0.78) and 0.11 for COWA (d = 0.14). To address missing post-intervention data, a missing completely random test was performed. Multiple imputations employing the predictive mean matching (PMM) method were used [32,33]. The dataset exhibited 2.5% missing data, which was addressed using multiple imputations via PMM to impute ten values.

Baseline descriptive characteristics were compared between groups using an independent *t*-test. Cognitive outcomes were analyzed using separate analysis of covariance (ANCOVA) tests, with change scores (post-intervention score minus baseline score) on the DSST and COWA. Each cognitive outcome was treated as a dependent variable, while the treatment group was the independent variable in the analysis. Covariates included in the ANCOVA tests were baseline scores of the dependent variable (to account for variance between groups), education, race, and age. Assumptions for ANCOVA were assessed prior to analysis. Linearity between covariates and dependent variables was confirmed through scatterplots. Homogeneity of the regression slopes was tested via an interaction model. Levene’s test verified homogeneity of variance across groups, and normality was assessed using the Shapiro–Wilk test. All assumptions were met. To account for the false discovery rate, we utilized a Tukey HSD test for post-intervention comparisons. Plots were generated utilizing z-scored cognitive change scores. The Spearman correlation test was used as a conservative approach to investigate the relationship between cognitive outcomes (DSST and COWA) with physical outcomes (SPPB and walking speed) within the intervention group. All analyses were conducted using RStudio version 2022.7.1.554.

## 3. Results

### 3.1. Participants

Initial descriptive characteristics for the total sample and groups are displayed in Table 1. No significant differences were found between groups for any baseline variables. In line with the study eligibility criteria, participants enrolled in the study were middle-aged or older women with overweight or obesity, identified as non-Hispanic Black or White, with functional limitations (as measured by SPPB), and self-reported a sedentary lifestyle [22]. Following the screening, 34 participants were randomized, with 17 participants being randomized to the EC group and 17 to the CR + E intervention group. Of the 34 participants who were initially enrolled in the study, two individuals dropped out of the study, including one from the CR + E group and one from the EC, with 32 participants completing the six-month study intervention. There was a total of two participant dropouts during the six-month intervention, one within the CR + E group due to personal health issues and one in the EC group due to scheduling conflicts.

### 3.2. Digit Symbol Substitution Test (DSST)

**Figure 1:**
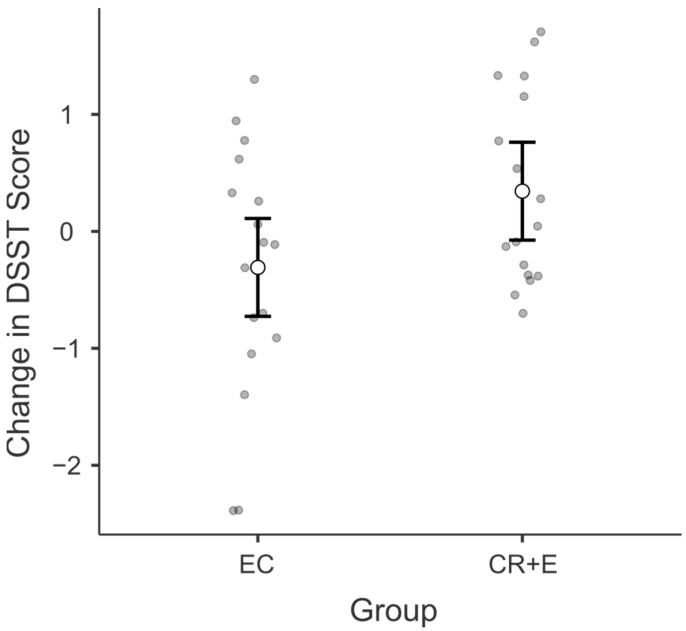
A figure representing the changes in group scores (z-scored) for the Digit Symbol Substitution Test (DSST). The figure incorporates error bars, denoting the 95% confidence interval. CR + E, caloric restriction plus exercise; EC, education control.

At post-intervention, there were significant differences in mean DSST scores between intervention groups (Figure 1). The overall model of the ANCOVA was significant [F (2, 28) = 3.70, *p* = 0.01]. The EC group exhibited a mean decrease in the DSST score of −1.06 points (SE 2.25), while the CR + E group showed a mean increase of 6.10 points (SE 2.25). There was a significant difference between the EC and the CR + E intervention groups with a positive moderate effect (Δ = −7.17, *p* = 0.03, d = 0.78).

### 3.3. Controlled Oral Word Association (COWA)

**Figure 2:**
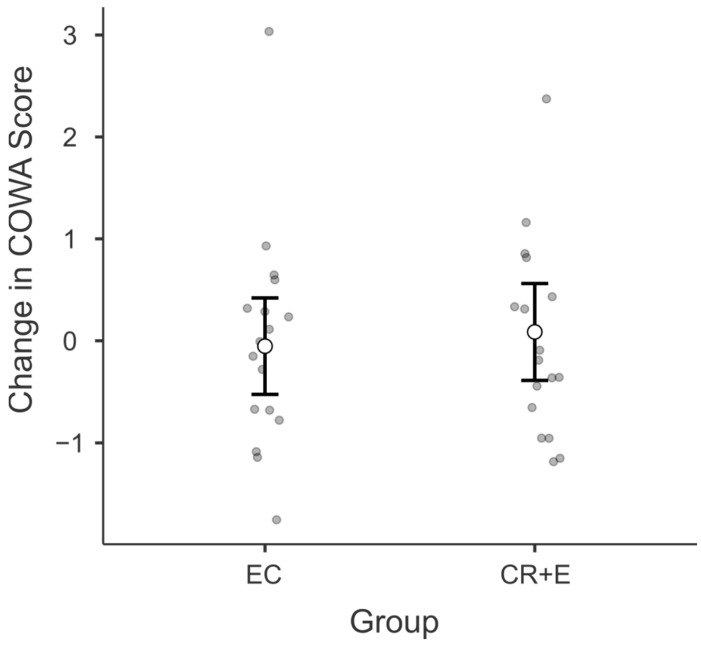
A figure representing the changes in group scores (z-scored) for the Controlled Oral Word Association (COWA) test. The figure incorporates error bars, denoting the 95% confidence interval. CR + E, caloric restriction plus exercise; EC, education control.

At post-intervention, there was no significant difference in COWA scores between groups (Figure 2). The overall model of the ANCOVA was non-significant [F (2, 28) = 1.76, *p* = 0.15]. Specifically, the EC group had a mean increase in COWA scores of 0.62 points (SE = 1.43), while the CR + E group showed a mean increase of 1.48 points (SE = 1.46).

### 3.4. Adherence, Weight Loss, and Physical Functioning

Those randomized to the CR + E group had 83% (SD 16%) and 70% (SD 26%) attendance at the weight loss and exercise group sessions, respectively. The parent study previously demonstrated significant changes between groups for weight and physical function. The CR + E group showed significantly greater weight loss (−5.72 kg; 95% CI: −8.6, −2.82) and improvements in walking speed (0.14 m/s; 95% CI: 0.04, 0.24) compared to the EC group. The CR + E group also showed significantly greater improvement in SPPB scores compared to the EC group (1.02; 95% CI: 0.16, 1.88; *p* = 0.02) [22].

### 3.5. Correlation Between Changes in Cognitive and Physical Function

**Figure 3:**
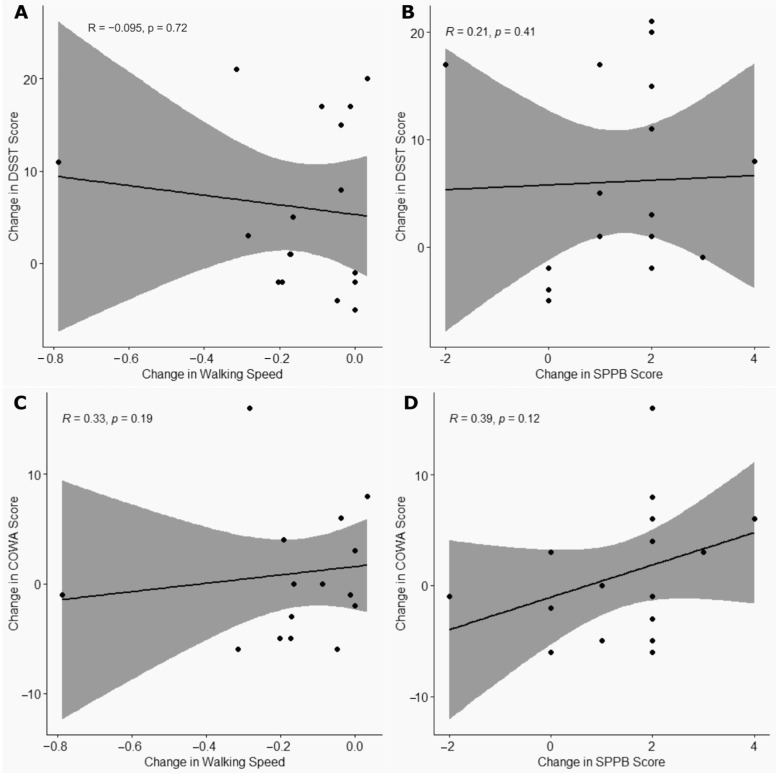
Correlations between changes observed in the caloric restriction plus exercise (CR + E) group: (**A**) Digit Symbol Substitution Test (DSST) score and walking speed, (**B**) DSST score and Short Physical Performance Battery (SPPB) score, (**C**) Controlled Oral Word Association (COWA) score and walking speed, and (**D**) COWA score and SPPB score.

No significant associations were observed between changes in DSST or COWA scores and changes in physical function for the CR + E group (Figure 3). The change in the DSST scores was not significantly correlated with changes in walking speed (rho = −0.01, *p* = 0.72) or SPPB scores (rho = 0.21, *p* = 0.11). Similarly, changes in COWA scores were not significantly associated with changes in walking speed (rho = 0.33, *p* = 0.19) or SPPB scores (rho = 0.39, *p* = 0.12).

## 4. Discussion

This study contributes to the literature by informing the effects of a combined CR + E intervention on cognitive function in a diverse sample of postmenopausal women with overweight or obesity and functional limitations. A significant group difference was observed in changes in complex attention, as measured by the DSST, with the CR + E group showing statistically significant improvements compared to the EC group. This effect was moderate in size and remained significant after adjusting for education, age, race, and baseline DSST score. In contrast, no significant group differences were observed for changes in language fluency, as measured by the COWA, after controlling for age, education, race, and baseline COWA score. Furthermore, for our exploratory aims, no significant associations were observed between changes in COWA or DSST with changes in SPPB scores or walking speed for the intervention group.

The significant increase in DSST scores from pre- to post-intervention provides preliminary data to suggest that 6 months of CR + E in a diverse sample of postmenopausal women with overweight or obesity and functional limitations may improve the cognitive domain of complex attention. The DSST is a well-established measure of cognitive function, and higher DSST scores have been positively correlated with the level of independence in adults [29]. This finding contributes to the existing body of evidence from observational studies in older adults, which indicate that improved diet quality or participation in structured exercise may independently enhance cognitive function, as measured by the DSST, in older adults [10,34]. Contrary to our findings, a randomized controlled trial investigating the effects of diet plus exercise, diet alone, or control in middle-aged men and women (mean age = 53 years) with hypertension showed no significant improvement in the DSST scores for the diet plus exercise group [26]. The reason for the mixed findings is unclear, but it may be attributed to differences in the populations studied (e.g., the age of participants) and variations in the intervention protocols, i.e., the shorter duration of four months compared to six months in our study and the exclusive focus on aerobic exercise compared to combined aerobic and anaerobic exercise used in our study.

While improvements were anticipated in both the COWA and DSST for the intervention group, significant gains were observed only in DSST scores, indicating enhancements in complex attention, which encompasses processing speed, sustained and divided attention, inhibition, and working memory [29]. Age-related biological and metabolic changes, such as central and peripheral impairments in glucose metabolism and inflammation, increase the risk of disability and cognitive decline and are strongly influenced by lifestyle factors like overnutrition and physical inactivity [35,36,37]. This is particularly important as obesity is increasingly linked to an elevated risk of cognitive decline, driven by a combination of biological, psychological, and social factors. Specifically, chronic inflammation and insulin resistance associated with obesity can lead to neuroinflammation, impacting executive function and memory, while vascular changes may exacerbate these cognitive impairments [38]. In addition to these biological factors, psychosocial elements such as weight stigma and social isolation may contribute to cognitive decline by increasing stress, disrupting sleep, fostering depression, and reducing physical activity, further heightening the risk of cognitive decline [39,40,41]. Thus, the CR + E intervention may have influenced DSST performance through improvements in insulin sensitivity and reductions in cerebral oxidative stress; however, these changes were not directly assessed in this study [42,43,44]. Furthermore, due to the nature of the group-based design, the intervention may have facilitated social connection and mental stimulation, both known to benefit cognitive function [45]. Many other biopsychosocial mechanisms exist, which may contribute to the observed improvements. Future studies are needed to elucidate the specific mechanisms involved.

In contrast to the improvements in DSST scores, we did not observe a statistical improvement in COWA scores from pre- to post-intervention for either the EC or CR + E group. This finding is consistent with a previously conducted RCT by Smith et al., which found that after a 4-month weight loss and exercise intervention in adults with hypertension, there were no significant changes in the score on the COWA test from pre- to post-intervention [26]. In contrast to our findings, Napoli and colleagues reported significant improvements in COWA scores after one year of combined diet and exercise intervention in older adults with obesity [24]. The differences in findings between studies may stem from the intervention length, one year in Napoli et al.’s study versus six months in ours, as well as variations in participant demographics, with their sample being older and more educated on average. Additionally, differences in language fluency measures, specifically semantic fluency (animal naming) in their study versus phonemic fluency (FAS test) in ours, could also explain some discrepancies.

Although prior research has shown a link between cognitive and physical function in aging adults, particularly those with functional limitations [10], our findings did not reveal a significant association between changes in cognitive and physical function within the CR + E group. We observed weak to moderate, but non-significant, correlations between changes in cognitive function and changes in physical function. These findings may reflect the limited sample size and exploratory nature of the analysis.

Although trends suggest a potential relationship, the lack of statistical significance also points to the possibility that other biopsychosocial mechanisms, unrelated to physical changes, may also contribute to cognitive improvements. The CR + E intervention may have influenced complex attention through pathways such as increased cognitive engagement, stress reduction, or social connectedness fostered by the group-based format, though these were not directly assessed in the study. These findings highlight the need to identify the mechanisms driving cognitive change.

Furthermore, the current study utilized a combined caloric restriction and exercise intervention, which limits the ability to determine the independent effects of either exercise or diet alone. While the question of which is more effective for weight loss is well established, a meta-analysis conducted by Johns et al. (2014) demonstrated that participants randomized to caloric restriction alone showed greater total weight loss compared to exercise alone, while the combination of caloric restriction and exercise resulted in the highest weight loss overall [46]. The combined approach remains the most efficient approach for weight reduction and improvements in cardiometabolic outcomes, while additionally promoting long-term weight maintenance in individuals with obesity [23,46,47]. However, less is known about whether exercise, a hypocaloric diet, or the combination produce differential outcomes related to cognitive function. A groundbreaking study by Napoli et al. recruited 107 participants who were randomized to a control group, exercise alone, caloric restriction alone, or a combined caloric restriction and exercise group; they found that those randomized to both the exercise alone and combined intervention showed more favorable increases in cognitive function compared to diet alone [24]. These findings suggest that while caloric restriction may be more effective for weight loss, using exercise independent of caloric restriction or with its combination may lead to greater improvements in cognitive outcomes.

While the preliminary results of the current study are promising, they should be interpreted in the context of the limitations of the study. Specifically, since the parent study focused on assessing the feasibility of a caloric restriction combined with exercise intervention in a diverse group of postmenopausal women, it was not adequately powered to detect more subtle effects. Consequently, the relatively small sample size may limit the detection of small to moderate effect sizes and potentially also result in inflated effect estimates, compared to studies with larger sample sizes. Additionally, the findings may not be generalizable beyond our specific demographic of educated middle-aged or older women with overweight or obesity, self-reported sedentary activity, and non-Hispanic Black or White racial identities. The study utilized participants’ self-reported energy intake and physical activity; thus, the assessment may be influenced by potential participant bias. Additionally, we implemented a standardized caloric deficit across participants, which limited the ability to individualize caloric prescriptions based on participants’ anthropometric characteristics. Furthermore, the study’s A/B design, with measurements taken at only two time points, limited our ability to observe temporal trends in cognitive outcomes. Our study outcomes were limited to the cognitive domains assessed by the changes in DSST and COWA. Additionally, we did not measure psychological factors, which may have been altered by group intervention. Finally, the combined nature of exercise and diet interventions prevents the identification of independent effects of these interventions.

The study also had several notable strengths. The inclusion of a racially diverse sample, with over fifty percent comprising non-Hispanic Black women, explores the effects of a combined intervention in a demographic at heightened risk. Additionally, the study implemented a comprehensive exercise regimen, encompassing both aerobic and resistance training, under the supervision of trained professionals, which not only ensured participant safety but also standardized exercise technique and intensity (6–20 Borg RPE scale). The choice of conducting intervention sessions at community centers reveals the practicality and scalability of this lifestyle-based approach within community settings. Furthermore, the utilization of widely accepted neuropsychological measures such as the DSST and COWA allows for these study findings to be repeated and compared to other studies, given the widespread accessibility across various research contexts and availability.

In future studies, larger sample sizes should be utilized to increase statistical power and precision in effect size estimation. Additionally, incorporating temporal interval measurements would enable the examination of both immediate and delayed effects of interventions, allowing for the facilitation of analysis of trends in change scores over time. Furthermore, utilizing a more comprehensive cognitive battery would offer a more thorough assessment of neuropsychological domains, and also integrating psychological measures, such as quality of life, depression, and weight-related stigma, would help identify potential confounding variables affected by CR + E. Future studies should use gold-standard methods to track energy intake and physical activity, such as photo-based food diary apps and actigraphy, as well as incorporating individualized caloric restriction and exercise recommendations. Lastly, the incorporation of multiple arms in future studies to assess the effects of dietary and exercise changes, both combined and independently, would enable the determination of independent and synergistic effects.

## 5. Conclusions

The present study found preliminary evidence to indicate that a combined intervention involving CR + E can improve complex attention, as measured by the DSST, in postmenopausal women with overweight or obesity and functional limitations. However, the results do not suggest a significant improvement in language fluency, as assessed by the COWA test. Additionally, the changes observed in the measured domains of cognitive function were not significantly related to changes in included measures of physical function. These preliminary findings suggest that CR + E interventions may enhance complex attention in an at-risk population of functionally limited postmenopausal women with overweight or obesity; however, longer studies with larger sample sizes are needed to confirm these preliminary findings.

## Figures and Tables

**Table 1:** Baseline characteristics for the total sample, educational control (EC) group, and caloric restriction plus exercise (CR + E) group.

Characteristics Mean [SD]	Total N = 34	EC N = 17	CR + E N = 17
Age (years)	63.7 [5.6]	63.7 [6.7]	63.7 [4.5]
Non-Hispanic Black	53%	53%	53%
BMI (kg/m^2^)	36.9 [6.2]	35.9 [6.8]	37.9 [5.5]
Education (years)	14.4 [2.7]	14.4 [3.1]	14.5 [2.3]
COWA	31.4 [9]	29.9 [8]	32.7 [9.9]
DSST	41.3 [11.1]	41.1 [10.2]	41.5 [12.1]
Walking Speed (m/s)	1 [0.3]	1 [0.3]	1 [0.3]
SPPB	9.2 [1]	9.1 [1.1]	9.3 [0.9]

Abbreviations: COWA, Controlled Oral Word Association; DSST, Digit Symbol Substitution Test; CR + E, Caloric Restriction plus Exercise; EC, Education Control; SPPB, Short Physical Performance Battery; BMI, body mass index; kg/m^2^, kilogram per meter squared; m/s, meters per second.

## Data Availability

The data used in this study are from a secondary analysis and have been previously cited elsewhere. The data is available on request from the corresponding author, subject to restrictions due to privacy and ethical considerations. For further details, please refer to the original citation.
